# The Impact of Neurophysiological Intraoperative Monitoring during Spinal Cord and Spine Surgery: A Critical Analysis of 121 Cases

**DOI:** 10.7759/cureus.1861

**Published:** 2017-11-19

**Authors:** Tarik Ibrahim, Oliver Mrowczynski, Omar Zalatimo, Vernon Chinchilli, Jonas Sheehan, Robert Harbaugh, Elias Rizk

**Affiliations:** 1 Department of Neurosurgery, Penn State Hershey Medical Center; 2 Department of Neurosurgery, Lifebridge Health - Sinai Hospital; 3 Public Health Sciences, Penn State University College of Medicine; 4 Neurosurgery, Holy spirit hospital

**Keywords:** intraoperative monitoring, spinal surgery, neuromonitoring, spine surgery

## Abstract

Neuromonitoring has been utilized during spinal surgery to assess the function of the spinal cord in an effort to prevent intraoperative injury. Although its use is widespread, no clear benefit has been demonstrated. Our goal in this study was to interrogate the value of intraoperative neuromonitoring in decreasing the severity and rate of neurological injury during and after spinal surgery. Here we describe our experience of 121 patients who underwent spinal cord procedures with the combination of intraoperative neuromonitoring, to determine its ability to detect neurological changes and the specificity and sensitivity in this setting. The data for the 121 patients who underwent neurophysiological monitoring during various spinal procedures was collected retrospectively. The patients were classified into one of four groups according to the findings of intraoperative monitoring and the clinical outcomes on postoperative neurological exam. Intraoperative monitoring was evaluated for its specificity, sensitivity, and predictive value. In our cohort of 121 patients, the use of intraoperative neuromonitoring had a low sensitivity, which may produce an excessive number of false negatives. Based on these findings, neuromonitoring seems to have a poor positive predictive value and is thus an inappropriate test to prevent harm to patients.

## Introduction

Neuromonitoring has long been used during spinal surgery to assess the function of the spinal cord in an effort to prevent intraoperative injury [1]. Although its use is widespread, no clear benefit has been demonstrated. Some evidence suggests that intraoperative monitoring is a cost-effective component of spinal surgery [2] that provides critical information enabling the surgical team to give the patient optimal postoperative neurologically functional outcomes. There are other reports in the literature that demonstrate a failure of neuromonitoring to predict postoperative outcome [3-8]. The exact efficacy of the utilization of intraoperative neuromonitoring is not well understood. Patient outcomes and improvement following surgical procedures is paramount, and testing whether intraoperative neuromonitoring aids in this regard is critical. Our goal in this study was to interrogate the value of intraoperative neuromonitoring to decrease the severity and rate of neurological injury during and after spinal surgery. Here we describe our experience of 121 patients who underwent spinal cord procedures utilizing intraoperative neuromonitoring, to determine its ability to be specific and sensitive for the accurate diagnosis of neurological deficit in this setting.

## Materials and methods

From January to December 2006, 121 patients (61 male, 60 female) underwent spinal surgery with multimodality intraoperative neurophysiologic monitoring. The ages ranged from one month old to 83 years old, with a mean age of 41.4 years. The cases were categorized into cervical, thoracic, and lumbar regions (Table 1).

**Table 1:** Summary of operative procedures

Spinal Region	Operation	N
Cervical (57)	Halo adjustment	1
	Corpectomy	2
	Anterior fusion	3
	Intramedullary tumor resection	2
	Laminectomy	9
	Posterior fusion	8
	ACDF	32
Thoracic (30)	Kyphoplasty	1
	Revision of spinal rods	1
	Anterior fusion	1
	Corpectomy	1
	Posterior fusion	5
	Laminectomy	7
	Thoraco-lumbar fusion	14
Lumbar (34)	Laminectomy	1
	Discectomy	1
	Pars repair	1
	Radical tumor resection	1
	Intramedulalry tumor resection	3
	Osteotomy/revision/lengthening of spinal rods	5
	Posterior fusion	22
Total (121)		

Fifty-seven cervical operations and seven types of cervical procedures were performed. These included halo-vest adjustment, anterior cervical discectomy and fusion (ACDF) with instrumentation, corpectomy and fusion with instrumentation, anterior fusion with instrumentation, posterior fusion with instrumentation (one of which extended to the thoracic spine), laminectomy for decompression, and intramedullary tumor resection. Thirty thoracic procedures were performed, including posterior thoracolumbar fusions, posterior fusion with instrumentation, corpectomy and instrumented fusion, laminectomy for decompression, kyphoplasty, and revision of spinal instrumentation and fusion. Thirty-four lumbar procedures were done. These included laminectomy, discectomy, posterior fusion with instrumentation, pars interarticularis fracture open reduction and internal fixation, intramedullary tumor resection, tethered cord release, and osteotomy revision with lengthening of spinal rods.

Neurophysiological potentials were monitored continuously throughout surgery. Modalities varied upon indications and included the following (with percentage of patients receiving each in parentheses): somatosensory evoked potentials (SSEPs; 98.4%), transcranial motor evoked potentials (TCMEPs; 86.3%), electromyography (EMG; 90.2%), train of four (TOF; 34.1%), electroencephalography (EEG; 19.5%), and brainstem auditory evoked responses (BAER; 1.6%).

Recording protocol

General anesthesia was induced in all cases with a mixture of propofol, fentanyl, and rocuronium. Anesthesia was thereafter maintained by propofol and fentanyl.

SSEPs

Median and ulnar nerve SSEPs (MN-SSEP and UN-SSEP, respectively) were elicited at the wrist via nerve stimulation for a duration of 300 msec and approximately 25 mA intensity at a 4.76 Hz stimulation rate. Posterior tibial nerve SSEPs (PT-SSEPs) were elicited at the ankle by the same parameters used in the upper extremities. The stimulation rate was sometimes lowered to 3.1 Hz if the baseline amplitudes were low. Surface pad electrodes were used to stimulate the SSEPs. The SSEPs were recorded using subcutaneous needle electrodes.

The recording arrays for MN-SSEPs and UN-SSEPs were placed at C3'/C4' with the reference electrode Fpz, Cv5'-Fpz. A proximal, brachial plexus recording was taken at RErb's point - LErb's point or the reverse depending on the side of stimulation. For the PT-SSEPs, the recording arrays are C3'/C4'-Fpz, C3'-C4' (or reverse depending on the side of stimulation) and Cv5-Fpz.

A surface pad ground electrode was placed on the deltoid or trapezius. The resistance of all electrodes was below 5 kOhm and all were within 2 kOhm of each other. The time base was set to 100 msec, filter settings at 10-3 kHz, amplifier gain at 100 uV/div and typically averaged between 200 and 300 trials.

BAEPs, aka auditory brainstem response (ABR)

The filter settings were 30-3k Hz with an amp gain of 10 uV/div. We used EAR-3 foam insert earphones with a distal stimulus generator and an air tube conducting the stimulus. The initial stimulation intensity was 70 dB SL, but if an audiogram was not available, the typical starting range for normal hearing patients was 80 dB HL. Rare fraction clicks were used at a rate between 11.1 and 33.1 Hz. Recording electrode arrays were A1/A2-Cz' and A1-A2 (or reverse depending on the stimulus side). Needle electrodes were placed in the ear canals. The time base was 20 msec and 1000-2000 trials were averaged. 

Stimulation, recording, and data processing were all done with the Cadwell Cascade (Cadwell Laboratories, Inc. WA, USA). The evoked potentials (EPs) were recorded and visually analyzed for the presence of the main peaks. For MN-SSEPs and UN-SSEPs, the cortical complex N20-P25 was identified. For the PT-SSEPs, the cortical complex of P37-N45 was analyzed. ABR waves I, III, and V were analyzed for presence or absence. Interpeak and I-V latencies were monitored. Wave V amplitude was also monitored. The baselines were collected post-induction, but pre-incision. Occasionally, if the patient was unstable, baselines were collected before the patient was positioned on the surgical table and then again after positioning. Data was then compared and the positioning was adjusted accordingly.

Collection of data

The criteria by which a significant neuromonitoring finding was defined were the following: MN-SSEPs: a reduction in amplitude by more than 50% in the cortical complex N20-P25 and/or an increase of the peak latency of N20 by more than 10% compared to the preoperative baseline; PT-SSEPs: a reduction in cortical complex P40-N50 amplitude of more than 50% and/or an increase of P40 peak latency by more than 10% compared to the baseline value. Loss of Waves I, III or V in any combination was considered a pathological finding for BAEP. When a neuromonitoring abnormality was detected, the surgeon and anesthesiologist were immediately notified and the following possible sources were immediately explored: physiological irregularities, anesthetic changes, and surgical manipulation.

Every patient had a neurological examination performed postoperatively by a member of the neurosurgery team. All deficits were documented and compared to the preoperative examination to determine if neurological findings were present preoperatively or acquired during surgery. Deficits were re-evaluated at the patient’s first outpatient visit following discharge and compared to the findings immediately following surgery. The neurological findings were correlated with intraoperative neuromonitoring changes. Based on this assessment, patients were assigned to one of four categories: false positive, true positive, false negative, and true negative. Patients who developed a neuromonitoring change that could not be corrected intraoperatively but who did not display any new neurological deficit postoperatively were designated as false positives. Patients who developed a neuromonitoring change intraoperatively and who demonstrated a new neurological deficit postoperatively were designated as true positives. In addition, a patient was designated as a true positive if an intraoperative neuromonitoring change occurred, the change was corrected intraoperatively, and the patient awoke without a new deficit. Patients who developed a new neurological deficit intraoperatively without a change in neuromonitoring were designated as false negatives. Finally, if no neuromonitoring changes occurred and the patient awoke without a new deficit, this was determined to be a true negative.

## Results

**Figure 1:**
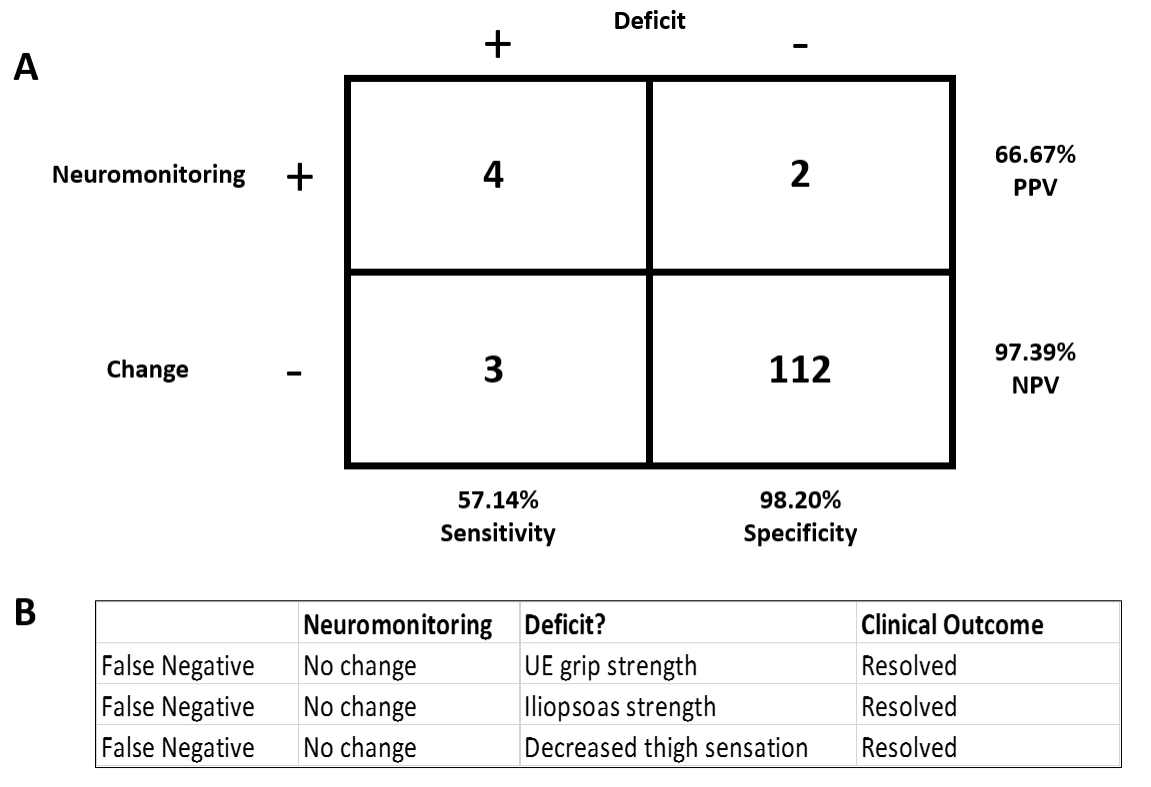
Results of our analysis on the use of intraoperative monitoring during 121 spinal surgery cases (A) Out of 121 patients, seven had neurological deficits. Four of those patients were accurately diagnosed with neuromonitoring, while the other three had false negatives. Out of the 114 patients who did not have a neurological deficit, neuromonitoring accurately diagnosed the lack  of deficits in 112 (Negative Predictive Value - NPV), while the other two had false positives (Positive Predictive Value - PPV). (B) The three patients who had clinically diagnosed neurological deficits in upper extremity (UE) grip strength, iliopsoas strength, and decreased thigh sensation were not accurately diagnosed by neuromonitoring.

We describe the results of 121 patients who underwent spinal surgeries as described in Table 1 with intraoperative neuromonitoring (Figure 1A).

Four out of seven patients who had a neurological deficit were able to be detected by neuromonitoring. Neuromonitoring was not able to detect the present deficits in the three other patients. This equates to a sensitivity of 57.14%. Out of 114 patients who did not have a neurological deficit, neuromonitoring accurately diagnosed no deficits in 112. The two other patients were falsely rendered as having deficits, when there were no clinical neurological deficits present, equating to a specificity of 98.20%. The positive predicted value of this data is 66.67%, while the negative predicted value is 97.39%.

We further assessed this data with regard to the three patients who were deemed having false negative results. The three patients had clinically diagnosed neurological deficits (Figure 1B). The first patient had an upper extremity deficit of grip strength at their postoperative check that was resolved by the next day. Another patient had iliopsoas strength graded at 4/5 immediately postoperatively, which improved to 5/5 at follow-up in the outpatient clinic. The third patient woke from surgery with decreased sensation on the antero-lateral portion of the thigh, which persisted until the first postoperative clinic appointment. These deficits were not accurately diagnosed by neuromonitoring.

## Discussion

Spinal surgery is a common procedure that has a risk of neurological deficit [9]. Damage to the spinal cord during a procedure can occur through stretch of the cord/nerve, hypoxia, or direct damage [10]. Intraoperative neuromonitoring is a tool with the goal of providing patients with limited morbidities and optimal outcomes during and after surgery. The aim of neuromonitoring during an operation is to provide the surgeon with a real-time analysis of spinal cord function at a time when there is still a possibility to correct any possibility of morbidity [11]. Changes in intraoperative neuromonitoring measurements can be due to changes in arterial pressure, cardiopulmonary function, and spinal cord function [12]. Potentials can also be influenced by anesthetic regimen [3, 13-15], perfusion pressure [16-17], hypothermia [18], and hyperthermia [19]. Intraoperative neuromonitoring has been utilized in many contexts, including spine surgery, arteriovenous malformations, thyroid and parathyroid surgery, pediatric deformity correction surgery, epilepsy surgery, subarachnoid hemorrhage repair, and others. Although it has been used in the numerous contexts shown above, an obvious benefit of intraoperative neuromonitoring providing optimal functional outcomes in patients has not been demonstrated.

One area highlighting this contention in the case of spinal surgery is spinal tumor resection. In a review by Scibilia, et al. on the use of intraoperative neuromonitoring in the specific scenario of spinal tumors, the authors came to the conclusion that neuromonitoring is a useful technology to aid in providing patients with adequate postoperative outcomes [20]. Other studies assessing the effect of neuromonitoring in spinal tumor surgery demonstrate that motor evoked potentials and multi-modal monitoring did not accurately provide predictive value for permanent functional deficits [21]. Significant changes occur most frequently in intramedullary lesions [22]. In such cases SSEPs have been found to have good sensitivity but poor specificity [23], and there are concerns that false positive changes during SSEP monitoring could also prematurely halt adequate surgical intervention [24]. Combining MEP with SSEP allows for a better prediction of motor outcome in spinal cord surgery. False positive results with MEPs have also occurred [25-26]. Furthermore, MEPs are highly variable and very sensitive to the effect of anesthesia and muscle relaxant. This adds another variable to the prediction of motor deficits through neuromonitoring [18]. Wiedmayer, et al. reported that in more than 50% of the cases, the surgeon was not able to respond to a monitoring event [22].

To try and enhance the efficacy of intraoperative neuromonitoring, “checklists” are being developed that provide a methodology of utilizing this technology optimally during surgery [27-28]. These checklists describe what the surgeon, anesthesiologist, neurophysiologists, and technicians should perform to manage a significant alert given by the intraoperative neuromonitoring [27].

The clinical goal of any test is to provide a result with a high degree of sensitivity and specificity with the hope of improving patient care and outcomes. When either of these attributes are low, data interpretation is confounded. In the case of intraoperative neuromonitoring, a low sensitivity was found in the present study, which may produce an excessive number of false negatives. Other studies such as that done by May, et al. found intraoperative monitoring to have low specificity such that a physician may experience more false positives [3]. Both low sensitivity and low specificity can have detrimental effects on the surgery and adversely affect patient outcomes. Particularly in cases in which there is a high risk of neurological injury, a lack of detected neurophysiological change (false negative) may give the surgeon a false sense of security and encourage him to continue performing a potentially damaging progress. The absence of findings could lead a surgeon to go beyond the bounds of his clinical and surgical judgment, exposing the patient to greater danger and harm.

The authors acknowledge several limitations of the present study. First, all included data was collected and reported retrospectively, thus some publication bias may exist. Second, there was a small sample size of patients who did have neurological deficits. Further studies in a greater number of patients are necessary to determine the full extent of efficacy of intraoperative neuromonitoring in the context of spine surgery.

## Conclusions

Our goal in this study was to interrogate the value of intraoperative neuromonitoring to decrease the severity and rate of neurological injury during and after spinal surgery. In our cohort of 121 patients, the use of intraoperative neuromonitoring had a low sensitivity, which may produce an excessive number of false negatives. Spine surgeons need to be aware of the low sensitivity and positive predictive value with neuromonitoring so that they rely more on their clinical and surgical judgement and interpret neuromonitoring with more scrutiny.
